# Evaluation of kidney dysfunction in childhood cancer survivors

**DOI:** 10.1038/s41390-022-02015-w

**Published:** 2022-03-25

**Authors:** Asmaa Abdel Sameea Mahmoud, Heba Badawy Abd Elsalam, Sara Mahmoud El-Deeb, Fouad Mohamed Zanaty, Hesham Mohamed Aboelghar, Mohamed Shokry Elharoun

**Affiliations:** 1 Department of Pediatrics, Faculty of Medicine, Menoufia University, Shebin Elkom, Egypt; 2 Department of Clinical Pathology, Faculty of Medicine, Menoufia University, Shebin Elkom, Egypt; 3 Department of Urology, Faculty of Medicine, Menoufia University, Shebin Elkom, Egypt

## Abstract

**Background:**

The major increase in the survival rate among children with cancer is due to improvement in the diagnosis and treatment. Despite this increase, childhood cancer survivors (CCS) are at high risk of developing late complications such as nephrotoxicity due to chemotherapy. So, we aimed to detect early subclinical kidney dysfunction among CCS.

**Methods:**

This cross-sectional study was implemented on 52 survivors of childhood cancer recruited from Pediatric Oncology Unit, Menoufia University. Laboratory evaluations for each participant, including complete blood count, serum urea, creatinine, urinary protein, urinary calcium, uric acid, and serum cystatin C and urinary Neutrophil Gelatinase Associated Lipocalin (UrNGAL) by ELISA were obtained.

**Results:**

Estimated GFR was decreased in 23.1% of cases, with elevated serum cystatin C, UrNGAL and UrNGAL/Cr. There was a significant increase of Uprotein/Cr, UCa/Cr, UACR (*p* = 0.02), UrNGAL and UrNGAL/Cr (*P* < 0.001) in patients with tubular dysfunction compared without tubular dysfunction. There was a significant difference between two groups regarding cisplatin (*P* = 0.03) and high-dose methotrexate chemotherapy (*p* = 0.04). The AUCs for detecting kidney tubular dysfunction by UrNGAL and UrNGAL/Cr were 0.807 and 0.747.

**Conclusion:**

A significant tubular dysfunction among childhood cancer survivors receiving chemotherapy as cisplatin and high-dose methotrexate.

**Impact:**

- Detection of kidney dysfunction mainly tubular in childhood cancer survivors after finishing chemotherapy.
- Urinary NGAL is a good predictor for detection of tubular dysfunction in childhood cancer survivors after finishing chemotherapy.

## Introduction

The survival rate among children with cancer was significantly improved as a result of anti-cancer treatment. 40% of these cancer patients suffer from growth impairment, neurotoxicity, heart failure, nephrotoxicity, hormonal disturbances and secondary cancer as late complications.^1^ Risk factors of nephrotoxicity include age, innate drug toxicity, anti-cancer treatment (chemotherapy, surgery, and radiation), and pre-existing kidney damage.^2^ The most common chemotherapy agents which are responsible for kidney injury are ifosfamide and cisplatin, also other anti-cancer drugs as high-dose methotrexate are associated with compromised glomerular function. Anti-cancer therapy for leukemia and lymphoma including high-dose methotrexate. Methotrexate toxicity acts mainly on the hematopoietic system and the intestinal mucosa.^3^ Kidney damage in CCS may be due to the precipitation of methotrexate and its less soluble metabolites (7-OH-MTX and DAMPA) in acidic urine.^4^

Cystatin- C due to its small size, is filtered by the glomerulus, reabsorbed and broken down by kidney tubules and involved in glomerular dysfunction.^5^

Urine neutrophil gelatinase-associated lipocalin (NGAL) is a protein that was extruded from injured proximal tubular cells into the urine. NGAL is involved in tubular cell injury and repair.^6^

Therefore, we aimed to estimate serum cystatin-C for detecting kidney glomerular dysfunction and urine NGAL for detecting kidney tubular dysfunction in survivors of pediatric cancer.

## Subjects and methods

### Design

A cross-sectional study was implemented on fifty-two survivors of pediatric cancer and was evaluated between May 2021 and September 2021. The 25 girls and 27 boys were 4–18 years of age at the time of evaluation; they were recruited from Oncology Unit, Pediatrics Department, Menoufia University. Prior to blood samples collection, a written informed consent was approved from the Ethics Committee of Faculty of Medicine, Menoufia University (ID: 24/5/2021.PEDI) was obtained from the guardians of all participants.

The participants were divided into 2 groups: Group I: included 25 patients with kidney tubular dysfunction. Group II: included 27 patients without kidney tubular dysfunction. Sociodemographic information, cancer type and treatment details, and specific antibacterial and antifungal drugs administered were abstracted from the medical records of each participant who underwent a complete physical examination.

## Methods

Laboratory investigations as complete blood count, serum urea, creatinine, calcium, potassium, sodium, and Cystatin C by ELISA were done. Urinary protein/creatinine ratio, calcium/creatinine ratio and urine uric acid were done through a spot test, and NGAL by ELISA.

Serum urea, creatinine, calcium, potassium, sodium were measured on the Beckman Coulter AU680 analyzer (Indianapolis, IN). Cystatin C was measured using the RayBio^®^ Human Cystatin C ELISA Kit supplied by RayBiotech* (Catalog #: ELH-Cystatin C, Raybiotech, Inc., Norcross, Georgia) according to manufacturer’s instructions. Detection Range: 0.3–20 ng/ml, standard curve points: 20, 10, 5, 2.5, 1.25, 0.625, 0.313 and 0 ng/ml, Intra-Assay CV%: <10% and Inter-Assay CV%: <12%. Urine NGAL was analyzed by an NGAL-ELISA kit (Kit 201-12-1720, Sunred Biological Technology Co., Ltd, Shanghai, China). Urinary calcium, uric acid and urinary calcium/creatinine ratio were measured using the Integra 800 device (Pisa, Italy). The estimated glomerular filtration rate (eGFR) was calculated using the modified Schwartz formula for children.^7^ A normal value of eGFR was ≥90 mL/min/1.73 m^2^ and the decreased value of eGFR was <90 mL/min/1.73 m^2^. Participants were defined to have tubular dysfunction if they had abnormal levels of UProtein/Cr, UCa/Cr, UACR, and UrNGAL.

### Statistical analysis

The primary outcome was the prevalence of tubular and glomerular dysfunction. The secondary outcome was the comparison between patients with and without tubular dysfunction by the tubular markers.

Data were analyzed using IBM SPSS statistics version 20 (SPSS Inc., Chicago, IL). Chi-square test was used to examine the relationship between qualitative variables. Fisher’s exact test was used when the expected cell count of more than 25% of cases was less than 5. For quantitative data, comparison between two groups was done using either student *t*-test or Mann–Whitney test (non-parametric *t*-test) as appropriate. Pearson’s correlation coefficient or Spearman-rho method (as appropriate) was used to test correlation between numerical variables. Receiver Operator Characteristic Curve (ROC) is a graphic representation of the relationship between sensitivity and specificity at different cut-off points for UrNGAL and UrNGAL/Cr. A *p* value <0.05 was considered significant.

## Results

### Demographic and clinical data of studied survivors

Fifty-two cases were recruited from the oncology unit. They were 25 girls and 27 boys, their ages ranged from 4 to 18 years old. 35 cases (67.3%) were diagnosed acute lymphoblastic leukemia, 9 cases (17.3%) were non-Hodgkin lymphoma, 2 cases (3.8%) were Hodgkin lymphoma, 3 cases (5.8%) were Neuroblastoma and 3 cases (5.8%) were Wilms tumor. Duration of chemotherapy ranged from 4 to 36 months according to type of cancer. Age at diagnosis of cancer ranged from 3.5 to 15 years old, age at follow up ranged from 4 to 18 years old. Elapsed time from diagnosis to follow-up ranged from 10 to 60 months. Duration after completing chemotherapy ranged from 4 to 24 months. 25 cases (48.1%) had tubular dysfunction and 27 cases (51.9%) had normal tubular function. There was a significant difference in the frequency of treatment with cisplatin and high-dose methotrexate chemotherapy between those with and without tubular dysfunction (Table 1).

**Table 1:** Demographic and clinical characteristics of all studied cases.

Demographic and clinical characteristics	Total studied cases (No. = 52)			
	Mean	Median	SD	Range
Age at diagnosis (years)	8.87	8	2.68	3.5–15
Age at follow up (years)	11.9	11	3.6	4–18
Elapsed time from diagnosis to follow-up (months)	45.7	50	9.8	10–60
Elapsed time from the end of treatment to follow up (months)	18.4	12	6.4	4–24
Sex				
Male	27 (51.9%)			
Female	25 (48.1%)			
Distribution of diagnosis				
Acute lymphoblastic leukemia	35 (67.3%)			
Neuroblastoma	3 (5.8%)			
Hodgkin lymphoma	2 (3.8%)			
Non-Hodgkin lymphoma	9 (17.3%)			
Wilms tumor	3 (5.8%)			
Duration of chemotherapy (months)	27.7	34	12.5	6–36

Type of chemotherapy	Cases with tubular dysfunction (No. = 25)	Cases without tubular dysfunction (No. = 27)	Test of significance	*P* value
Cisplatin	No %	No %		
Yes	4 (16.0%)	5 (18.5%)	*χ*^2^ test=4.67	0.03*
No	21 (84.0%)	22(81.0%)		
Methotrexate				
Yes	24 (96.0%)	25 (92.6%)	*χ*^2^test = 4.38	0.04*
No	1 (4.0%)	2 (7.4%)		
Cytarabine				
Yes	19 (76.0%)	22 (81.5%)	*χ*^2^ test = 0.23	0.63
No	6 (24.0%)	5 (18.5%)		
Cyclophosphsmide			Fisher’s exact test=1.93	
Yes	25 (100%)	25 (92.6%)		0.49
No	0 (0.0%)	2 (7.4%)		

*SD* standard deviation.

*significant difference <0.05.

Mild anemia was recorded in some cases. Serum electrolytes (Na, K, and Ca), serum urea and creatinine were normal in all patients. eGFR was decreased in 12 cases, and serum Cystatin C was increased in the same 12 cases who had decreased eGFR. The urine calcium/creatinine ratio (UCa/Cr), urine protein/creatinine ratio (Uprotein/Cr) and urine albumin/creatinine ratio were increased in 25 cases (48.1%). In addition, elevated levels of UrNGAL in 30 cases (57.7%) including 5 cases without tubular dysfunction who had a mild increase in UrNGAL and elevated levels of UrNGAL/Cr in all cases (100%) as shown in (Table 2).

**Table 2:** Laboratory findings of all studied cases.

Laboratory findings	Total studied cases (No. = 52)			
	Mean	Median	SD	Range
Hb (gm/dl)	12.8	12.8	1.4	10–16
WBCs (×10^3^/mm^3^)	6.3	5.8	2.6	4–13.7
**P**latelet ((×10^3^/mm^3^)	250.7	241	71.4	160–452
Na (mEq/L)	135.3	135	1.5	134–139
K (mEq/L)	4.1	4	0.31	3.7–5
Ca (mg/dl)	9.2	9	0.54	8.5–10.6
Serum urea (mg/dl)	22.5	23	4.1	12–28
Serum creatinine (mg/dl)	0.79	0.80	0.12	0.6–1.1
eGFR (mL/min./1.73m^2^)	104.5	100	17.8	75–135 (12 cases <90)
Uprotein/Cr	0.33	0.38	0.09	0.12–0.46 (25 cases >0.25)
UACR (mg/g)	45	25	17.3	10–75 (25 cases >30)
UCa/Cr	0.28	0.32	0.12	0.15–0.45 (25 cases >0.22)
Uric acid in urine. Normal value (250–750 mg/24 h)	340.8	320	140.5	160–700
Serum cystatin C (mg/L)	122.6	112.5	57.4	40–300
UrNGAL (ng/ml)	1989.9	1983.5	488.4	1061–3114
UrNGAL/Cr	2598.6	2437.5	738.1	1178–3974

*Hb* Hemoglobin, *WBCs* white blood cells, *Na* sodium, *K* potassium, *Ca* calcium, *eGFR* estimated glomerular filtration rate, *UACR* urinary albumin creatinine ratio, *NGAL* neutrophil gelatinase-associated lipocalin.

### Comparison between cases with tubular dysfunction and those without tubular dysfunction

There was a significant difference in Uprotein/Cr (*P* < 0.001), UCa/Cr (*P* < 0.001), UrNGAL (*P* < 0.001) and UrNGAL/Cr (*P* < 0.001) in patients with tubular dysfunction compared to those without tubular dysfunction (Table 3).

**Table 3:** Comparison between cases with tubular dysfunction and those without tubular dysfunction regarding urinary markers.

Parameters	Total cases (No. = 52)		Test of significance	*P* value
	With tubular dysfunction (No. = 25)	Without tubular dysfunction (No. = 27)		
Uprotein/Cr Median (range) Normal value up to 0.2 mg/mg	0.35 (0.25–0.46)	0.13 (0.09–0.22)	_________	<0.001**
UCa/Cr Median (range) Normal value up to 0.22 mg/mg	0.31 (0.28–0.45)	0.16 (0.12–0.22)	_________	<0.001**
UACR (mg/g) Normal value 2–30 mg/g	43.87 ± 17.98	31.93 ± 14.57	*t*-test = 3.22	0.02
UrNGAL (ng/ml) Normal value 0.2–132				
Mean ± SD	2249.3 ± 497.4	174.9 ± 33.91	*t*-test = 4.19	<0.001**
UrNGAL/Cr Normal value 1.3–16.3				
Mean ± SD	2990.9 ± 747.4	223.53 ± 51.76	*t*-test = 4.21	<0.001**

*significant difference.

**Fig. 1:**
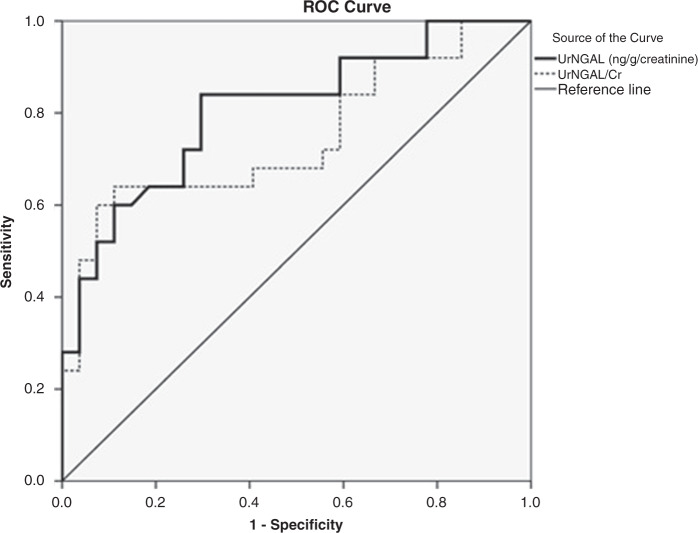
ROC curve of UrNGAL and UrNGAL/Cr for detection of Kidney tubular dysfunction.

There was a significant positive correlation between UrNGAL, and UrNGAL/Cr regarding serum urea (*r* = 0.307, *P* = 0.001, and *r* = 0.504, *P* < 0.001 respectively). Also, there was a significant positive correlation between serum cystatin C, UrNGAL, and UrNGAL/Cr regarding serum creatinine (*r* = 0.521, *P* < 0.001, *r* = 0.423, *P* = 0.003 and *r* = 0.494, *P* < 0.001 respectively). There was a significant positive correlation between UrNGAL, UrNGAL/Cr regarding Uprotein/Cr (*r* = 0.431, *P* = 0.001, and *r* = 0.408, *P* = 0.003 respectively). There was a significant positive correlation between UrNGAL and UACR (*r* = 0.472, *P* < 0.001). On the other hand, there was a significant negative correlation between serum Cystatin C regarding eGFR (*r* = −0.518, *P* < 0.001) as illustrated in (Table 4). Figure 1 and Table 5 showed the comparison of the diagnostic values between UrNGAL and UrNGAL/Cr for detection of kidney tubular dysfunction among studied cases using the ROC curve. The AUC of distinguishing patients with kidney tubular dysfunction from those without tubular dysfunction by UrNGAL was 0.807 at the cutoff point of 1964.7 ng/g/creatinine where the sensitivity was 84% with the specificity being 70.4%, the accuracy was 76.9%, the PPV was 72.4% and the NPV was 82.6%. The AUC of distinguishing patients with kidney tubular dysfunction from those without tubular dysfunction by UrNGAL/Cr was 0.747 at the cutoff value of 2182 at which level the sensitivity was 84% at a specificity of 40.7%, the accuracy was 61.5%, the PPV was 56.8% and the NPV was 73.3%.

**Table 4:** Correlation between serum Cystatin C, UrNGAL, UrNGAL/Cr and other parameters.

Parameters	Serum Cystatin C		UrNGAL (ng/ml)		UrNGAL/Cr	
	*r*	*P* value	*r*	*P* value	*r*	*P* value
Age (years)	−0.052	0.13	−0.300	0.03	−0.217	0.12
Duration of chemotherapy (months)	0.046	0.14	0.087	0.54	0.146	0.30
Na (mEq/L)	−0.147	0.42	−0.127	0.37	−0.189	0.18
K (mEq/L)	0.242	0.62	0.020	0.89	−0.066	0.64
Ca (mg/dl)	0.065	0.53	−0.055	0.69	0.199	0.16
Serum urea (mg/dl)	0.313	0.03	0.307	0.001^**^	0.504	<0.001**
Serum creatinine (mg/dl)	0.521	<0.001**	0.423	0.003^**^	0.494	<0.001**
eGFR (mL/min./1.73m^2^)	−0.518	<0.001**	−0.289	0.04	0.189	0.18
Uprotein/Cr	0.114	0.24	0.431	0.001**	0.408	0.003*
UACR (mg/g)	0.054	0.66	0.472	<0.001**	0.381	0.005
UCa/Cr	0.882	0.71	0.312	0.02	0.333	0.01
Uric acid in urine	0.212	0.16	0.335	0.01	0.306	0.03

*significant difference.

**Table 5:** ROC curve of UrNGAL and, UrNGAL/Cr for detection of Kidney tubular dysfunction.

Parameter	AUC	Cutoff point	Sensitivity	Specificity	PPV	NPV	Accuracy
UrNGAL	0.807	1964.7	84%	70.4%	72.4%	82.6%	76.9%
UrNGAL/Cr	0.747	2182	84%	40.7%	56.8%	73.3%	61.5%

*AUC* area under the curve, *PPV* positive predictive value, *NPV* negative predictive value.

## Discussion

The reported prevalence of kidney dysfunction varied from 0 to 84%. This wide range may be related to variations in the definition of abnormal kidney function and cohort factors, including the type of malignancy, chemotherapeutic agents, radiation therapy, and supportive drug use.^8–10^

The present study revealed tubular dysfunction among childhood cancer survivors. We evaluated patients by eGFR, proteinuria, albuminuria and urinary markers for tubular dysfunction. We found low eGFR in 12 cases (23.1%), proteinuria, microalbuminuria and elevated UCa/Cr, UrNGAL and UrNGAL/Cr as well as elevated serum Cystatin C. In addition, there was a significant difference between patients with tubular dysfunction and those without tubular dysfunction regarding cisplatin and methotrexate chemotherapy. Erdem et al.^11^ showed that low GFR was detected in 32% of all survivors mainly who received nephrotoxic drugs including aminoglycosides, vancomycin or amphotericin B. Certain studies revealed that cisplatin, high-dose methotrexate, and nephrotoxic drugs taken during febrile neutropenia were associated with low eGFR.^12–14^

A Cumulative dose of high-dose MTX, in the range of 1000–33,000 mg/m^2^ with a combination of calcium leucovorin, is associated with acute kidney injury (AKI) in 0–12.4% with an overall incidence of 1.8%.^15^

The current study revealed 36.5% of cases had proteinuria and 28.8% of cases had microalbuminuria. Erdem et al.^11^ showed that microalbuminuria was found in 10.1% of all survivors. Also; Oberlin et al.^16^ illustrated that proteinuria in 24-h urine collection was detected in 11.3% of survivors and Kninjenburg et al.^17^ detected albuminuria in 14.5% and decrease eGFR in 62 survivors (4.2%) of all 1442 survivors who were treated with chemotherapeutic.

The  current study showed elevated levels of UrNGAL, UrNGAL/Cr and serum Cystatin. Mehdiabadi et al.^18^ reported an abnormal increase in UrNGAL in 8.9% of patients with acute lymphoblastic leukemia with a mean level of 63 ± 113 ng/mL. In addition, eGFR was less than 60 mL/min/1.73 m^2^ in 13.3% of these patients indicating kidney dysfunction. Sterling et al.^19^ showed increased UrNGAL and IL-18 after cisplatin indicating AKI. Latoch et al.^20^ reported elevated UrNGAL/Cr ratio in cancer survivors receiving cisplatin and ifosfamide, cyclophosphamide and methotrexate. Also, they reported a significantly increased urinary NGAL, NGAL/Cr ratio and KIM-1/Cr ratio in solid tumor survivors indicating kidney tubular dysfunction many years after chemotherapy. Li et al.^21^ estimated the utility of serum NGAL in early detection of AKI induced by a high-dose of methotrexate.

Barnfield et al.^22^ reported a significant increase in serum Cystatin C and illustrated the diagnostic value of serum Cystatin C-assessed GFR that was significantly superior to that of serum creatinine-assessed GFR in the detection of kidney dysfunction in cancer children. Lankisch et al.^23^ revealed that serum Cystatin C was an alternative to serum creatinine for monitoring kidney functions and GFR in pediatric cancer patients with solid tumors and hematological malignancies as it is less dependent on influential factors like weight, height, and muscle mass. Serum Cystatin C had an increased diagnostic accuracy for decreased GFR when compared to serum creatinine.^23^

### Limitation of the study

Small sample size and a small number of cases receiving ifosfamide and cisplatin.

## Conclusion

There was a significant tubular dysfunction among childhood cancer survivors receiving chemotherapy as cisplatin and high-dose methotrexate. So, early detection of subclinical kidney dysfunction is very important for early intervention to prevent long-term complications such as chronic kidney disease.
